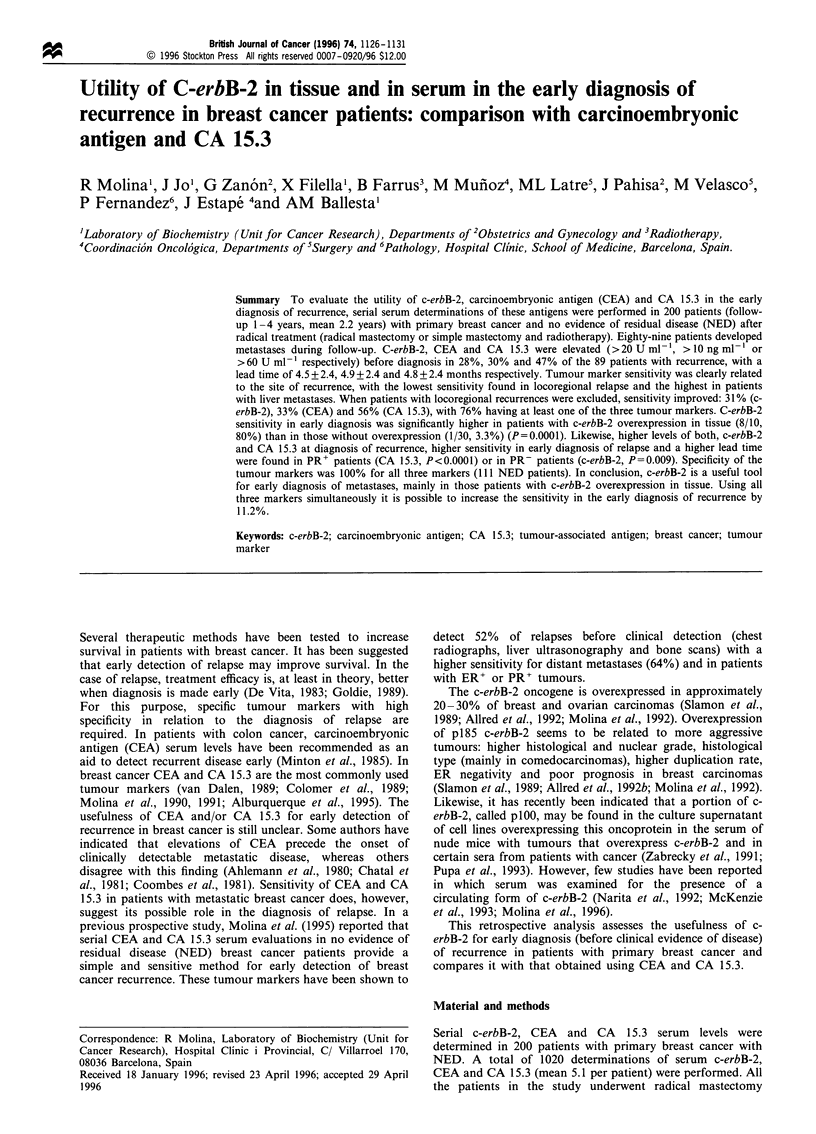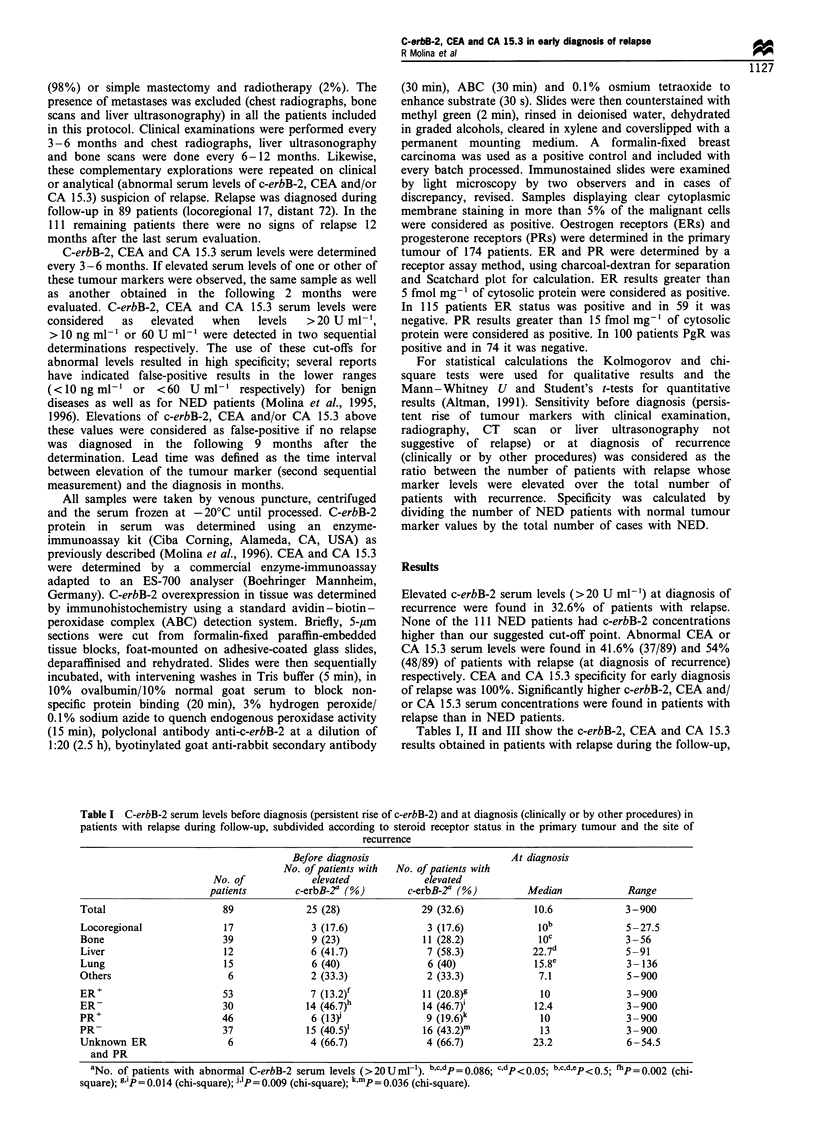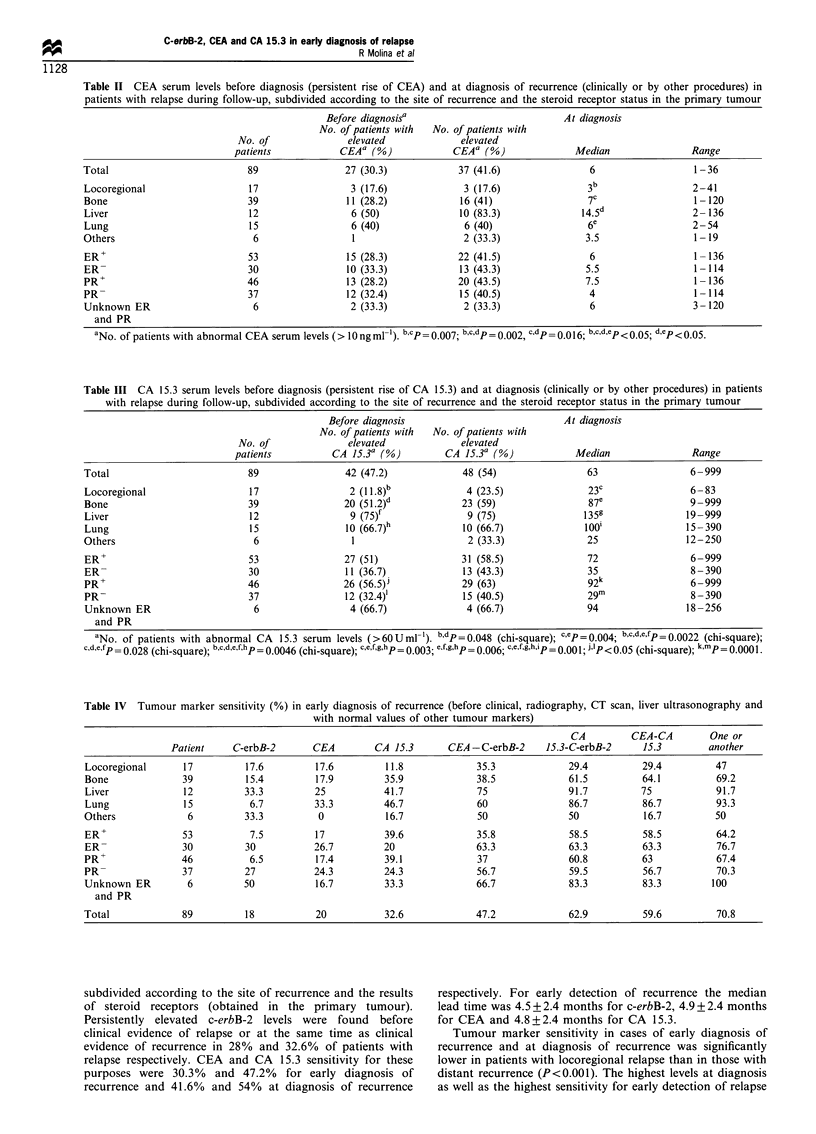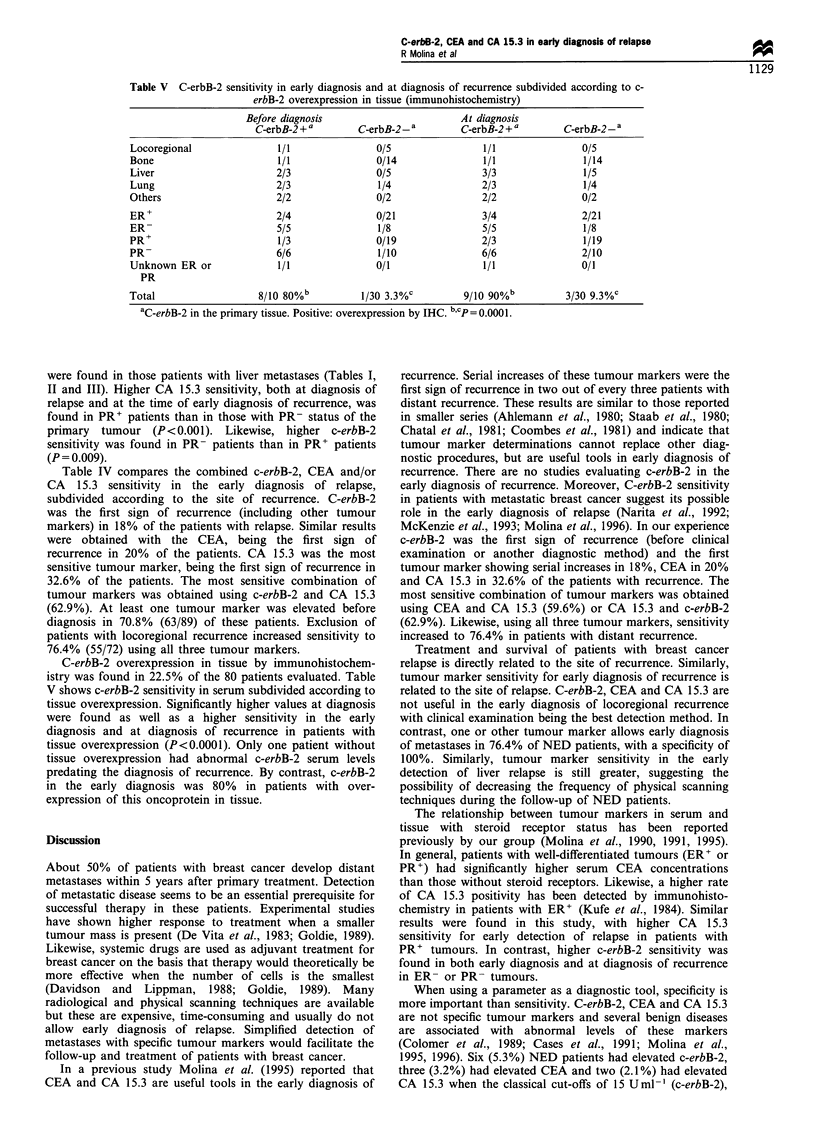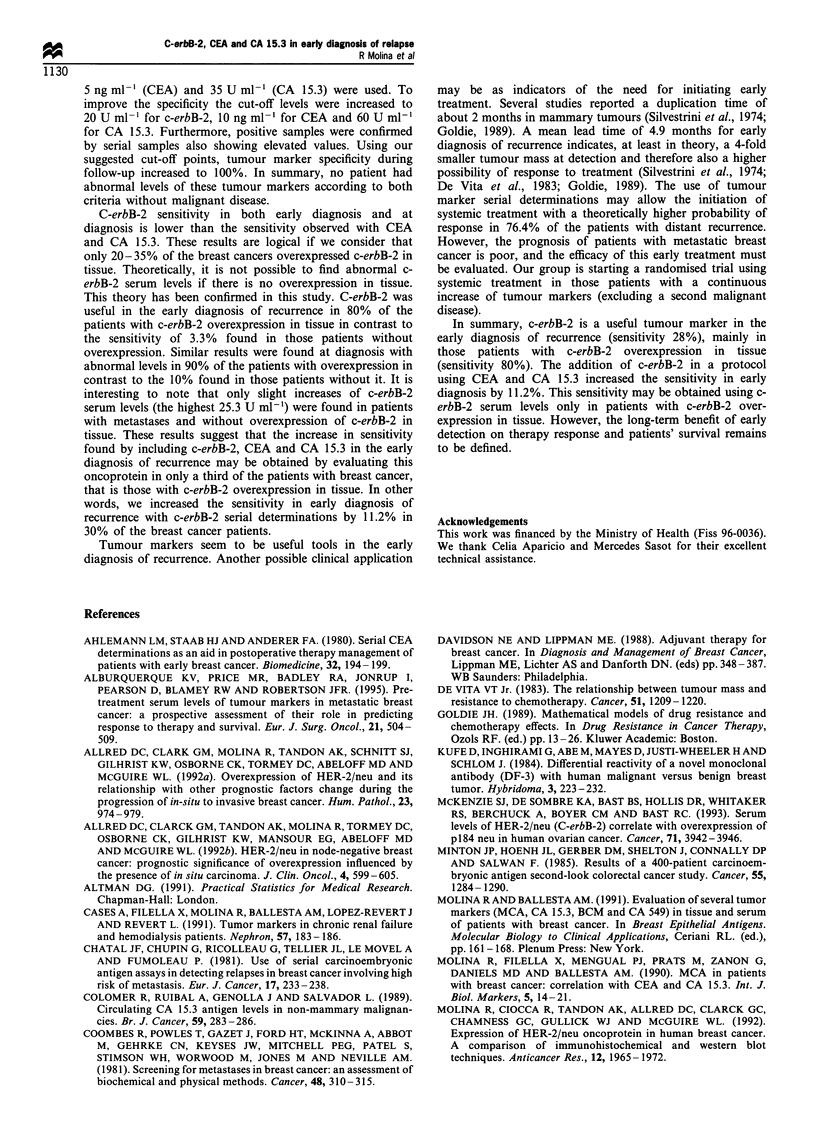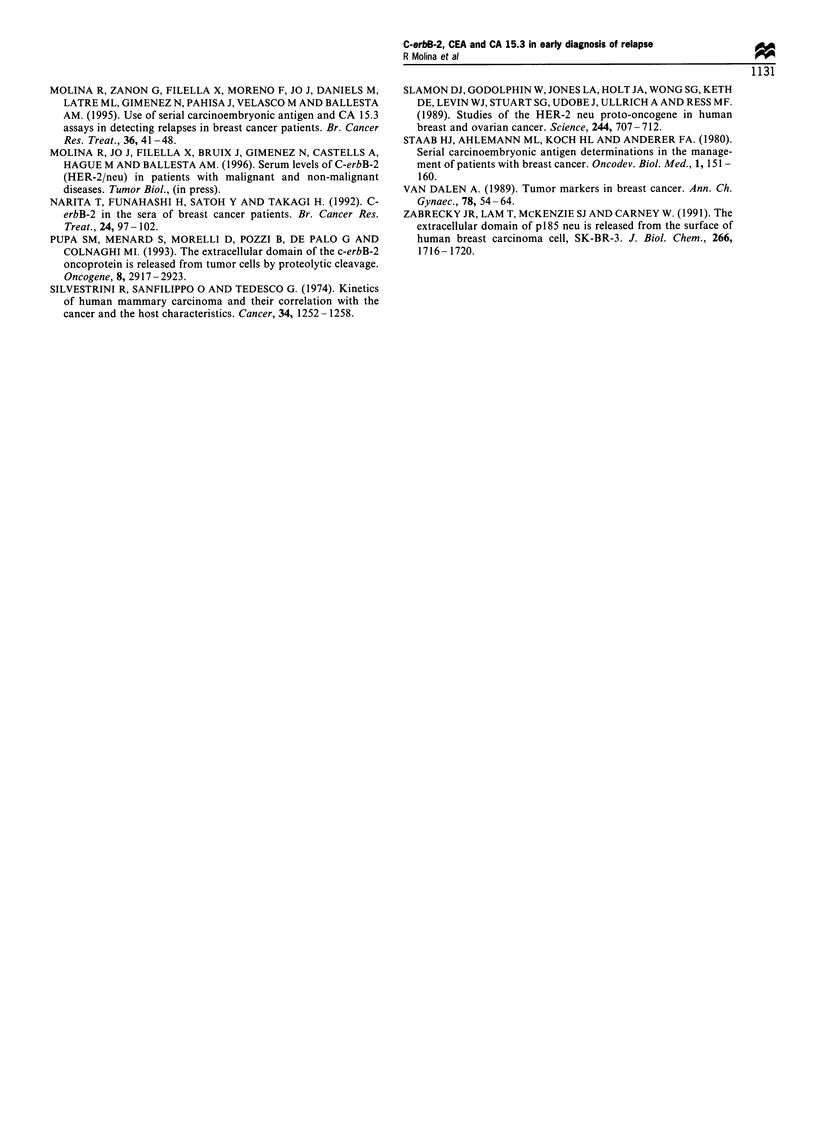# Utility of C-erbB-2 in tissue and in serum in the early diagnosis of recurrence in breast cancer patients: comparison with carcinoembryonic antigen and CA 15.3.

**DOI:** 10.1038/bjc.1996.501

**Published:** 1996-10

**Authors:** R. Molina, J. Jo, G. Zanón, X. Filella, B. Farrus, M. Muñoz, M. L. Latre, J. Pahisa, M. Velasco, P. Fernandez, J. Estapé, A. M. Ballesta

**Affiliations:** Laboratory of Biochemistry (Unit for Cancer Research), Hospital Clínic, School of Medicine, Barcelona, Spain.

## Abstract

To evaluate the utility of c-erbB-2, carcinoembryonic antigen (CEA) and CA 15.3 in the early diagnosis of recurrence, serial serum determinations of these antigens were performed in 200 patients (follow-up 1-4 years, mean 2.2 years) with primary breast cancer and no evidence of residual disease (NED) after radical treatment (radical mastectomy or simple mastectomy and radiotherapy). Eighty-nine patients developed metastases during follow-up. C-erbB-2, CEA and CA 15.3 were elevated (> 20 U ml-1, > 10 ng ml-1 or > 60 U ml-1 respectively) before diagnosis in 28%, 30% and 47% of the 89 patients with recurrence, with a lead time of 4.5 +/- 2.4, 4.9 +/- 2.4 and 4.8 +/- 2.4 months respectively. Tumour marker sensitivity was clearly related to the site of recurrence, with the lowest sensitivity found in locoregional relapse and the highest in patients with liver metastases. When patients with locoregional recurrences were excluded, sensitivity improved: 31% (c-erbB-2), 33% (CEA) and 56% (CA 15.3), with 76% having at least one of the three tumour markers. C-erbB-2 sensitivity in early diagnosis was significantly higher in patients with c-erbB-2 overexpression in tissue (8/10, 80%) than in those without overexpression (1/30, 3.3%) (P = 0.0001). Likewise, higher levels of both, c-erbB-2 and CA 15.3 at diagnosis of recurrence, higher sensitivity in early diagnosis of relapse and a higher lead time were found in PR+ patients (CA 15.3, P < 0.0001) or in PR- patients (c-erbB-2, P = 0.009). Specificity of the tumour markers was 100% for all three markers (111 NED patients). In conclusion, c-erbB-2 is a useful tool for early diagnosis of metastases, mainly in those patients with c-erbB-2 overexpression in tissue. Using all three markers simultaneously it is possible to increase the sensitivity in the early diagnosis of recurrence by 11.2%.


British Journal of Cancer (1996) 74, 1126–1131
© 1996 Stockton Press All rights reserved 0007–0920/96 $12.00

### Utility of C-*erb*B-2 in tissue and in serum in the early diagnosis of recurrence in breast cancer patients: comparison with carcinoembryonic antigen and CA 15.3

R Molina[1], J Jo[1], G Zanón[2], X Filella[1], B Farrus[3], M Muñoz[4], ML Latre[5], J Pahisa[2], M Velasco[5], P Fernandez[6], J Estapé [4]and AM Ballesta[1]

[1]*Laboratory of Biochemistry (Unit for Cancer Research), Departments of* [2]*Obstetrics and Gynecology and* [3]*Radiotherapy,* [4]*Coordinación Oncológica, Departments of* [5]*Surgery and* [6]*Pathology, Hospital Clinic, School of Medicine, Barcelona, Spain.*

**Summary** To evaluate the utility of c-*erb*B-2, carcinoembryonic antigen (CEA) and CA 15.3 in the early diagnosis of recurrence, serial serum determinations of these antigens were performed in 200 patients (follow-up 1–4 years, mean 2.2 years) with primary breast cancer and no evidence of residual disease (NED) after radical treatment (radical mastectomy or simple mastectomy and radiotherapy). Eighty-nine patients developed metastases during follow-up. C-*erb*B-2, CEA and CA 15.3 were elevated ($>20$ U $ml^{-1}$, $>10$ ng $ml^{-1}$ or $>60$ U $ml^{-1}$ respectively) before diagnosis in 28%, 30% and 47% of the 89 patients with recurrence, with a lead time of $4.5 \pm 2.4$, $4.9 \pm 2.4$ and $4.8 \pm 2.4$ months respectively. Tumour marker sensitivity was clearly related to the site of recurrence, with the lowest sensitivity found in locoregional relapse and the highest in patients with liver metastases. When patients with locoregional recurrences were excluded, sensitivity improved: 31% (c-*erb*B-2), 33% (CEA) and 56% (CA 15.3), with 76% having at least one of the three tumour markers. C-*erb*B-2 sensitivity in early diagnosis was significantly higher in patients with c-*erb*B-2 overexpression in tissue (8/10, 80%) than in those without overexpression (1/30, 3.3%) ($P=0.0001$). Likewise, higher levels of both, c-*erb*B-2 and CA 15.3 at diagnosis of recurrence, higher sensitivity in early diagnosis of relapse and a higher lead time were found in $PR^+$ patients (CA 15.3, $P<0.0001$) or in $PR^-$ patients (c-*erb*B-2, $P=0.009$). Specificity of the tumour markers was 100% for all three markers (111 NED patients). In conclusion, c-*erb*B-2 is a useful tool for early diagnosis of metastases, mainly in those patients with c-*erb*B-2 overexpression in tissue. Using all three markers simultaneously it is possible to increase the sensitivity in the early diagnosis of recurrence by 11.2%.

**Keywords:** c-*erb*B-2; carcinoembryonic antigen; CA 15.3; tumour-associated antigen; breast cancer; tumour marker

Several therapeutic methods have been tested to increase survival in patients with breast cancer. It has been suggested that early detection of relapse may improve survival. In the case of relapse, treatment efficacy is, at least in theory, better when diagnosis is made early (De Vita, 1983; Goldie, 1989). For this purpose, specific tumour markers with high specificity in relation to the diagnosis of relapse are required. In patients with colon cancer, carcinoembryonic antigen (CEA) serum levels have been recommended as an aid to detect recurrent disease early (Minton *et al.*, 1985). In breast cancer CEA and CA 15.3 are the most commonly used tumour markers (van Dalen, 1989; Colomer *et al.*, 1989; Molina *et al.*, 1990, 1991; Alburquerque *et al.*, 1995). The usefulness of CEA and/or CA 15.3 for early detection of recurrence in breast cancer is still unclear. Some authors have indicated that elevations of CEA precede the onset of clinically detectable metastatic disease, whereas others disagree with this finding (Ahlemann *et al.*, 1980; Chatal *et al.*, 1981; Coombes *et al.*, 1981). Sensitivity of CEA and CA 15.3 in patients with metastatic breast cancer does, however, suggest its possible role in the diagnosis of relapse. In a previous prospective study, Molina *et al.* (1995) reported that serial CEA and CA 15.3 serum evaluations in no evidence of residual disease (NED) breast cancer patients provide a simple and sensitive method for early detection of breast cancer recurrence. These tumour markers have been shown to detect 52% of relapses before clinical detection (chest radiographs, liver ultrasonography and bone scans) with a higher sensitivity for distant metastases (64%) and in patients with $ER^+$ or $PR^+$ tumours.

The c-*erb*B-2 oncogene is overexpressed in approximately 20–30% of breast and ovarian carcinomas (Slamon *et al.*, 1989; Allred *et al.*, 1992; Molina *et al.*, 1992). Overexpression of p185 c-*erb*B-2 seems to be related to more aggressive tumours: higher histological and nuclear grade, histological type (mainly in comedocarcinomas), higher duplication rate, ER negativity and poor prognosis in breast carcinomas (Slamon *et al.*, 1989; Allred *et al.*, 1992*b*; Molina *et al.*, 1992). Likewise, it has recently been indicated that a portion of c-*erb*B-2, called p100, may be found in the culture supernatant of cell lines overexpressing this oncoprotein in the serum of nude mice with tumours that overexpress c-*erb*B-2 and in certain sera from patients with cancer (Zabrecky *et al.*, 1991; Pupa *et al.*, 1993). However, few studies have been reported in which serum was examined for the presence of a circulating form of c-*erb*B-2 (Narita *et al.*, 1992; McKenzie *et al.*, 1993; Molina *et al.*, 1996).

This retrospective analysis assesses the usefulness of c-*erb*B-2 for early diagnosis (before clinical evidence of disease) of recurrence in patients with primary breast cancer and compares it with that obtained using CEA and CA 15.3.

### Material and methods

Serial c-*erb*B-2, CEA and CA 15.3 serum levels were determined in 200 patients with primary breast cancer with NED. A total of 1020 determinations of serum c-*erb*B-2, CEA and CA 15.3 (mean 5.1 per patient) were performed. All the patients in the study underwent radical mastectomy

Correspondence: R Molina, Laboratory of Biochemistry (Unit for Cancer Research), Hospital Clínic i Provincial, C/ Villarroel 170, 08036 Barcelona, Spain
Received 18 January 1996; revised 23 April 1996; accepted 29 April 1996

(98%) or simple mastectomy and radiotherapy (2%). The presence of metastases was excluded (chest radiographs, bone scans and liver ultrasonography) in all the patients included in this protocol. Clinical examinations were performed every 3–6 months and chest radiographs, liver ultrasonography and bone scans were done every 6–12 months. Likewise, these complementary explorations were repeated on clinical or analytical (abnormal serum levels of c-*erb*B-2, CEA and/or CA 15.3) suspicion of relapse. Relapse was diagnosed during follow-up in 89 patients (locoregional 17, distant 72). In the 111 remaining patients there were no signs of relapse 12 months after the last serum evaluation.

C-*erb*B-2, CEA and CA 15.3 serum levels were determined every 3–6 months. If elevated serum levels of one or other of these tumour markers were observed, the same sample as well as another obtained in the following 2 months were evaluated. C-*erb*B-2, CEA and CA 15.3 serum levels were considered as elevated when levels $>20$ U $ml^{-1}$, $>10$ ng $ml^{-1}$ or 60 U $ml^{-1}$ were detected in two sequential determinations respectively. The use of these cut-offs for abnormal levels resulted in high specificity; several reports have indicated false-positive results in the lower ranges ($<10$ ng $ml^{-1}$ or $<60$ U $ml^{-1}$ respectively) for benign diseases as well as for NED patients (Molina *et al*., 1995, 1996). Elevations of c-*erb*B-2, CEA and/or CA 15.3 above these values were considered as false-positive if no relapse was diagnosed in the following 9 months after the determination. Lead time was defined as the time interval between elevation of the tumour marker (second sequential measurement) and the diagnosis in months.

All samples were taken by venous puncture, centrifuged and the serum frozen at −20°C until processed. C-*erb*B-2 protein in serum was determined using an enzyme-immunoassay kit (Ciba Corning, Alameda, CA, USA) as previously described (Molina *et al*., 1996). CEA and CA 15.3 were determined by a commercial enzyme-immunoassay adapted to an ES-700 analyser (Boehringer Mannheim, Germany). C-*erb*B-2 overexpression in tissue was determined by immunohistochemistry using a standard avidin–biotin–peroxidase complex (ABC) detection system. Briefly, 5-μm sections were cut from formalin-fixed paraffin-embedded tissue blocks, foat-mounted on adhesive-coated glass slides, deparaffinised and rehydrated. Slides were then sequentially incubated, with intervening washes in Tris buffer (5 min), in 10% ovalbumin/10% normal goat serum to block non-specific protein binding (20 min), 3% hydrogen peroxide/0.1% sodium azide to quench endogenous peroxidase activity (15 min), polyclonal antibody anti-c-*erb*B-2 at a dilution of 1:20 (2.5 h), byotinylated goat anti-rabbit secondary antibody (30 min), ABC (30 min) and 0.1% osmium tetraoxide to enhance substrate (30 s). Slides were then counterstained with methyl green (2 min), rinsed in deionised water, dehydrated in graded alcohols, cleared in xylene and coverslipped with a permanent mounting medium. A formalin-fixed breast carcinoma was used as a positive control and included with every batch processed. Immunostained slides were examined by light microscopy by two observers and in cases of discrepancy, revised. Samples displaying clear cytoplasmic membrane staining in more than 5% of the malignant cells were considered as positive. Oestrogen receptors (ERs) and progesterone receptors (PRs) were determined in the primary tumour of 174 patients. ER and PR were determined by a receptor assay method, using charcoal-dextran for separation and Scatchard plot for calculation. ER results greater than 5 fmol $mg^{-1}$ of cytosolic protein were considered as positive. In 115 patients ER status was positive and in 59 it was negative. PR results greater than 15 fmol $mg^{-1}$ of cytosolic protein were considered as positive. In 100 patients PgR was positive and in 74 it was negative.

For statistical calculations the Kolmogorov and chi-square tests were used for qualitative results and the Mann–Whitney *U* and Student's *t*-tests for quantitative results (Altman, 1991). Sensitivity before diagnosis (persistent rise of tumour markers with clinical examination, radiography, CT scan or liver ultrasonography not suggestive of relapse) or at diagnosis of recurrence (clinically or by other procedures) was considered as the ratio between the number of patients with relapse whose marker levels were elevated over the total number of patients with recurrence. Specificity was calculated by dividing the number of NED patients with normal tumour marker values by the total number of cases with NED.

### Results

Elevated c-*erb*B-2 serum levels ($>20$ U $ml^{-1}$) at diagnosis of recurrence were found in 32.6% of patients with relapse. None of the 111 NED patients had c-*erb*B-2 concentrations higher than our suggested cut-off point. Abnormal CEA or CA 15.3 serum levels were found in 41.6% (37/89) and 54% (48/89) of patients with relapse (at diagnosis of recurrence) respectively. CEA and CA 15.3 specificity for early diagnosis of relapse was 100%. Significantly higher c-*erb*B-2, CEA and/or CA 15.3 serum concentrations were found in patients with relapse than in NED patients.

Tables I, II and III show the c-*erb*B-2, CEA and CA 15.3 results obtained in patients with relapse during the follow-up,

**Table I** C-*erb*B-2 serum levels before diagnosis (persistent rise of c-*erb*B-2) and at diagnosis (clinically or by other procedures) in patients with relapse during follow-up, subdivided according to steroid receptor status in the primary tumour and the site of recurrence

| | | Before diagnosis | At diagnosis | | |
|---|---|---|---|---|---|
| | No. of patients | No. of patients with elevated c-erbB-2[a] (%) | No. of patients with elevated c-erbB-2[a] (%) | Median | Range |
| Total | 89 | 25 (28) | 29 (32.6) | 10.6 | 3–900 |
| Locoregional | 17 | 3 (17.6) | 3 (17.6) | 10[b] | 5–27.5 |
| Bone | 39 | 9 (23) | 11 (28.2) | 10[c] | 3–56 |
| Liver | 12 | 6 (41.7) | 7 (58.3) | 22.7[d] | 5–91 |
| Lung | 15 | 6 (40) | 6 (40) | 15.8[e] | 3–136 |
| Others | 6 | 2 (33.3) | 2 (33.3) | 7.1 | 5–900 |
| $ER^+$ | 53 | 7 (13.2)[f] | 11 (20.8)[g] | 10 | 3–900 |
| $ER^-$ | 30 | 14 (46.7)[h] | 14 (46.7)[i] | 12.4 | 3–900 |
| $PR^+$ | 46 | 6 (13)[j] | 9 (19.6)[k] | 10 | 3–900 |
| $PR^-$ | 37 | 15 (40.5)[l] | 16 (43.2)[m] | 13 | 3–900 |
| Unknown ER and PR | 6 | 4 (66.7) | 4 (66.7) | 23.2 | 6–54.5 |

[a]No. of patients with abnormal C-*erb*B-2 serum levels ($>20$ U $ml^{-1}$). [b,c,d]$P=0.086$; [c,d]$P<0.05$; [b,c,d,e]$P<0.5$; [fh]$P=0.002$ (chi-square); [g,i]$P=0.014$ (chi-square); [j,l]$P=0.009$ (chi-square); [k,m]$P=0.036$ (chi-square).

**Table II** CEA serum levels before diagnosis (persistent rise of CEA) and at diagnosis of recurrence (clinically or by other procedures) in patients with relapse during follow-up, subdivided according to the site of recurrence and the steroid receptor status in the primary tumour

| | *No. of patients* | *Before diagnosis*[a] *No. of patients with elevated CEA*[a] *(%)* | *At diagnosis* *No. of patients with elevated CEA*[a] *(%)* | *Median* | *Range* |
|---|---|---|---|---|---|
| Total | 89 | 27 (30.3) | 37 (41.6) | 6 | 1–36 |
| Locoregional | 17 | 3 (17.6) | 3 (17.6) | 3[b] | 2–41 |
| Bone | 39 | 11 (28.2) | 16 (41) | 7[c] | 1–120 |
| Liver | 12 | 6 (50) | 10 (83.3) | 14.5[d] | 2–136 |
| Lung | 15 | 6 (40) | 6 (40) | 6[e] | 2–54 |
| Others | 6 | 1 | 2 (33.3) | 3.5 | 1–19 |
| $ER^+$ | 53 | 15 (28.3) | 22 (41.5) | 6 | 1–136 |
| $ER^-$ | 30 | 10 (33.3) | 13 (43.3) | 5.5 | 1–114 |
| $PR^+$ | 46 | 13 (28.2) | 20 (43.5) | 7.5 | 1–136 |
| $PR^-$ | 37 | 12 (32.4) | 15 (40.5) | 4 | 1–114 |
| Unknown ER and PR | 6 | 2 (33.3) | 2 (33.3) | 6 | 3–120 |

[a]No. of patients with abnormal CEA serum levels ($>10\,ng\,ml^{-1}$). [b,c]$P=0.007$; [b,c,d]$P=0.002$, [c,d]$P=0.016$; [b,c,d,e]$P<0.05$; [d,e]$P<0.05$.

**Table III** CA 15.3 serum levels before diagnosis (persistent rise of CA 15.3) and at diagnosis (clinically or by other procedures) in patients with relapse during follow-up, subdivided according to the site of recurrence and the steroid receptor status in the primary tumour

| | *No. of patients* | *Before diagnosis* *No. of patients with elevated CA 15.3*[a] *(%)* | *At diagnosis* *No. of patients with elevated CA 15.3*[a] *(%)* | *Median* | *Range* |
|---|---|---|---|---|---|
| Total | 89 | 42 (47.2) | 48 (54) | 63 | 6–999 |
| Locoregional | 17 | 2 (11.8)[b] | 4 (23.5) | 23[c] | 6–83 |
| Bone | 39 | 20 (51.2)[d] | 23 (59) | 87[e] | 9–999 |
| Liver | 12 | 9 (75)[f] | 9 (75) | 135[g] | 19–999 |
| Lung | 15 | 10 (66.7)[h] | 10 (66.7) | 100[i] | 15–390 |
| Others | 6 | 1 | 2 (33.3) | 25 | 12–250 |
| $ER^+$ | 53 | 27 (51) | 31 (58.5) | 72 | 6–999 |
| $ER^-$ | 30 | 11 (36.7) | 13 (43.3) | 35 | 8–390 |
| $PR^+$ | 46 | 26 (56.5)[j] | 29 (63) | 92[k] | 6–999 |
| $PR^-$ | 37 | 12 (32.4)[l] | 15 (40.5) | 29[m] | 8–390 |
| Unknown ER and PR | 6 | 4 (66.7) | 4 (66.7) | 94 | 18–256 |

[a]No. of patients with abnormal CA 15.3 serum levels ($>60\,U\,ml^{-1}$). [b,d]$P=0.048$ (chi-square); [c,e]$P=0.004$; [b,c,d,e,f]$P=0.0022$ (chi-square); [c,d,e,f]$P=0.028$ (chi-square); [b,c,d,e,f,h]$P=0.0046$ (chi-square); [c,e,f,g,h]$P=0.003$; [e,f,g,h]$P=0.006$; [c,e,f,g,h,i]$P=0.001$; [j,l]$P<0.05$ (chi-square); [k,m]$P=0.0001$.

**Table IV** Tumour marker sensitivity (%) in early diagnosis of recurrence (before clinical, radiography, CT scan, liver ultrasonography and with normal values of other tumour markers)

| | *Patient* | *C-*erb*B-2* | *CEA* | *CA 15.3* | *CEA–*C-erb*B-2* | *CA 15.3-C-*erb*B-2* | *CEA-CA 15.3* | *One or another* |
|---|---|---|---|---|---|---|---|---|
| Locoregional | 17 | 17.6 | 17.6 | 11.8 | 35.3 | 29.4 | 29.4 | 47 |
| Bone | 39 | 15.4 | 17.9 | 35.9 | 38.5 | 61.5 | 64.1 | 69.2 |
| Liver | 12 | 33.3 | 25 | 41.7 | 75 | 91.7 | 75 | 91.7 |
| Lung | 15 | 6.7 | 33.3 | 46.7 | 60 | 86.7 | 86.7 | 93.3 |
| Others | 6 | 33.3 | 0 | 16.7 | 50 | 50 | 16.7 | 50 |
| $ER^+$ | 53 | 7.5 | 17 | 39.6 | 35.8 | 58.5 | 58.5 | 64.2 |
| $ER^-$ | 30 | 30 | 26.7 | 20 | 63.3 | 63.3 | 63.3 | 76.7 |
| $PR^+$ | 46 | 6.5 | 17.4 | 39.1 | 37 | 60.8 | 63 | 67.4 |
| $PR^-$ | 37 | 27 | 24.3 | 24.3 | 56.7 | 59.5 | 56.7 | 70.3 |
| Unknown ER and PR | 6 | 50 | 16.7 | 33.3 | 66.7 | 83.3 | 83.3 | 100 |
| Total | 89 | 18 | 20 | 32.6 | 47.2 | 62.9 | 59.6 | 70.8 |

subdivided according to the site of recurrence and the results of steroid receptors (obtained in the primary tumour). Persistently elevated c-*erb*B-2 levels were found before clinical evidence of relapse or at the same time as clinical evidence of recurrence in 28% and 32.6% of patients with relapse respectively. CEA and CA 15.3 sensitivity for these purposes were 30.3% and 47.2% for early diagnosis of recurrence and 41.6% and 54% at diagnosis of recurrence respectively. For early detection of recurrence the median lead time was $4.5\pm2.4$ months for c-*erb*B-2, $4.9\pm2.4$ months for CEA and $4.8\pm2.4$ months for CA 15.3.

Tumour marker sensitivity in cases of early diagnosis of recurrence and at diagnosis of recurrence was significantly lower in patients with locoregional relapse than in those with distant recurrence ($P<0.001$). The highest levels at diagnosis as well as the highest sensitivity for early detection of relapse

**Table V** C-erbB-2 sensitivity in early diagnosis and at diagnosis of recurrence subdivided according to c-*erb*B-2 overexpression in tissue (immunohistochemistry)

| | *Before diagnosis* C-erb*B-2* +[a] | C-erb*B-2* −[a] | *At diagnosis* C-erb*B-2* +[a] | C-erb*B-2* −[a] |
|---|---|---|---|---|
| Locoregional | 1/1 | 0/5 | 1/1 | 0/5 |
| Bone | 1/1 | 0/14 | 1/1 | 1/14 |
| Liver | 2/3 | 0/5 | 3/3 | 1/5 |
| Lung | 2/3 | 1/4 | 2/3 | 1/4 |
| Others | 2/2 | 0/2 | 2/2 | 0/2 |
| $ER^+$ | 2/4 | 0/21 | 3/4 | 2/21 |
| $ER^-$ | 5/5 | 1/8 | 5/5 | 1/8 |
| $PR^+$ | 1/3 | 0/19 | 2/3 | 1/19 |
| $PR^-$ | 6/6 | 1/10 | 6/6 | 2/10 |
| Unknown ER or PR | 1/1 | 0/1 | 1/1 | 0/1 |
| Total | 8/10 80%[b] | 1/30 3.3%[c] | 9/10 90%[b] | 3/30 9.3%[c] |

[a]C-*erb*B-2 in the primary tissue. Positive: overexpression by IHC. [b,c]$P$=0.0001.

were found in those patients with liver metastases (Tables I, II and III). Higher CA 15.3 sensitivity, both at diagnosis of relapse and at the time of early diagnosis of recurrence, was found in $PR^+$ patients than in those with $PR^-$ status of the primary tumour ($P<0.001$). Likewise, higher c-*erb*B-2 sensitivity was found in $PR^-$ patients than in $PR^+$ patients ($P=0.009$).

Table IV compares the combined c-*erb*B-2, CEA and/or CA 15.3 sensitivity in the early diagnosis of relapse, subdivided according to the site of recurrence. C-*erb*B-2 was the first sign of recurrence (including other tumour markers) in 18% of the patients with relapse. Similar results were obtained with the CEA, being the first sign of recurrence in 20% of the patients. CA 15.3 was the most sensitive tumour marker, being the first sign of recurrence in 32.6% of the patients. The most sensitive combination of tumour markers was obtained using c-*erb*B-2 and CA 15.3 (62.9%). At least one tumour marker was elevated before diagnosis in 70.8% (63/89) of these patients. Exclusion of patients with locoregional recurrence increased sensitivity to 76.4% (55/72) using all three tumour markers.

C-*erb*B-2 overexpression in tissue by immunohistochemistry was found in 22.5% of the 80 patients evaluated. Table V shows c-*erb*B-2 sensitivity in serum subdivided according to tissue overexpression. Significantly higher values at diagnosis were found as well as a higher sensitivity in the early diagnosis and at diagnosis of recurrence in patients with tissue overexpression ($P<0.0001$). Only one patient without tissue overexpression had abnormal c-*erb*B-2 serum levels predating the diagnosis of recurrence. By contrast, c-*erb*B-2 in the early diagnosis was 80% in patients with overexpression of this oncoprotein in tissue.

### Discussion

About 50% of patients with breast cancer develop distant metastases within 5 years after primary treatment. Detection of metastatic disease seems to be an essential prerequisite for successful therapy in these patients. Experimental studies have shown higher response to treatment when a smaller tumour mass is present (De Vita *et al.*, 1983; Goldie, 1989). Likewise, systemic drugs are used as adjuvant treatment for breast cancer on the basis that therapy would theoretically be more effective when the number of cells is the smallest (Davidson and Lippman, 1988; Goldie, 1989). Many radiological and physical scanning techniques are available but these are expensive, time-consuming and usually do not allow early diagnosis of relapse. Simplified detection of metastases with specific tumour markers would facilitate the follow-up and treatment of patients with breast cancer.

In a previous study Molina *et al.* (1995) reported that CEA and CA 15.3 are useful tools in the early diagnosis of recurrence. Serial increases of these tumour markers were the first sign of recurrence in two out of every three patients with distant recurrence. These results are similar to those reported in smaller series (Ahlemann *et al.*, 1980; Staab *et al.*, 1980; Chatal *et al.*, 1981; Coombes *et al.*, 1981) and indicate that tumour marker determinations cannot replace other diagnostic procedures, but are useful tools in early diagnosis of recurrence. There are no studies evaluating c-*erb*B-2 in the early diagnosis of recurrence. Moreover, C-*erb*B-2 sensitivity in patients with metastatic breast cancer suggest its possible role in the early diagnosis of relapse (Narita *et al.*, 1992; McKenzie *et al.*, 1993; Molina *et al.*, 1996). In our experience c-*erb*B-2 was the first sign of recurrence (before clinical examination or another diagnostic method) and the first tumour marker showing serial increases in 18%, CEA in 20% and CA 15.3 in 32.6% of the patients with recurrence. The most sensitive combination of tumour markers was obtained using CEA and CA 15.3 (59.6%) or CA 15.3 and c-*erb*B-2 (62.9%). Likewise, using all three tumour markers, sensitivity increased to 76.4% in patients with distant recurrence.

Treatment and survival of patients with breast cancer relapse is directly related to the site of recurrence. Similarly, tumour marker sensitivity for early diagnosis of recurrence is related to the site of relapse. C-*erb*B-2, CEA and CA 15.3 are not useful in the early diagnosis of locoregional recurrence with clinical examination being the best detection method. In contrast, one or other tumour marker allows early diagnosis of metastases in 76.4% of NED patients, with a specificity of 100%. Similarly, tumour marker sensitivity in the early detection of liver relapse is still greater, suggesting the possibility of decreasing the frequency of physical scanning techniques during the follow-up of NED patients.

The relationship between tumour markers in serum and tissue with steroid receptor status has been reported previously by our group (Molina *et al.*, 1990, 1991, 1995). In general, patients with well-differentiated tumours ($ER^+$ or $PR^+$) had significantly higher serum CEA concentrations than those without steroid receptors. Likewise, a higher rate of CA 15.3 positivity has been detected by immunohistochemistry in patients with $ER^+$ (Kufe *et al.*, 1984). Similar results were found in this study, with higher CA 15.3 sensitivity for early detection of relapse in patients with $PR^+$ tumours. In contrast, higher c-*erb*B-2 sensitivity was found in both early diagnosis and at diagnosis of recurrence in $ER^-$ or $PR^-$ tumours.

When using a parameter as a diagnostic tool, specificity is more important than sensitivity. C-*erb*B-2, CEA and CA 15.3 are not specific tumour markers and several benign diseases are associated with abnormal levels of these markers (Colomer *et al.*, 1989; Cases *et al.*, 1991; Molina *et al.*, 1995, 1996). Six (5.3%) NED patients had elevated c-*erb*B-2, three (3.2%) had elevated CEA and two (2.1%) had elevated CA 15.3 when the classical cut-offs of 15 U $ml^{-1}$ (c-*erb*B-2),

5 ng $ml^{-1}$ (CEA) and 35 U $ml^{-1}$ (CA 15.3) were used. To improve the specificity the cut-off levels were increased to 20 U $ml^{-1}$ for c-*erb*B-2, 10 ng $ml^{-1}$ for CEA and 60 U $ml^{-1}$ for CA 15.3. Furthermore, positive samples were confirmed by serial samples also showing elevated values. Using our suggested cut-off points, tumour marker specificity during follow-up increased to 100%. In summary, no patient had abnormal levels of these tumour markers according to both criteria without malignant disease.

C-*erb*B-2 sensitivity in both early diagnosis and at diagnosis is lower than the sensitivity observed with CEA and CA 15.3. These results are logical if we consider that only 20–35% of the breast cancers overexpressed c-*erb*B-2 in tissue. Theoretically, it is not possible to find abnormal c-*erb*B-2 serum levels if there is no overexpression in tissue. This theory has been confirmed in this study. C-*erb*B-2 was useful in the early diagnosis of recurrence in 80% of the patients with c-*erb*B-2 overexpression in tissue in contrast to the sensitivity of 3.3% found in those patients without overexpression. Similar results were found at diagnosis with abnormal levels in 90% of the patients with overexpression in contrast to the 10% found in those patients without it. It is interesting to note that only slight increases of c-*erb*B-2 serum levels (the highest 25.3 U $ml^{-1}$) were found in patients with metastases and without overexpression of c-*erb*B-2 in tissue. These results suggest that the increase in sensitivity found by including c-*erb*B-2, CEA and CA 15.3 in the early diagnosis of recurrence may be obtained by evaluating this oncoprotein in only a third of the patients with breast cancer, that is those with c-*erb*B-2 overexpression in tissue. In other words, we increased the sensitivity in early diagnosis of recurrence with c-*erb*B-2 serial determinations by 11.2% in 30% of the breast cancer patients.

Tumour markers seem to be useful tools in the early diagnosis of recurrence. Another possible clinical application may be as indicators of the need for initiating early treatment. Several studies reported a duplication time of about 2 months in mammary tumours (Silvestrini *et al.*, 1974; Goldie, 1989). A mean lead time of 4.9 months for early diagnosis of recurrence indicates, at least in theory, a 4-fold smaller tumour mass at detection and therefore also a higher possibility of response to treatment (Silvestrini *et al.*, 1974; De Vita *et al.*, 1983; Goldie, 1989). The use of tumour marker serial determinations may allow the initiation of systemic treatment with a theoretically higher probability of response in 76.4% of the patients with distant recurrence. However, the prognosis of patients with metastatic breast cancer is poor, and the efficacy of this early treatment must be evaluated. Our group is starting a randomised trial using systemic treatment in those patients with a continuous increase of tumour markers (excluding a second malignant disease).

In summary, c-*erb*B-2 is a useful tumour marker in the early diagnosis of recurrence (sensitivity 28%), mainly in those patients with c-*erb*B-2 overexpression in tissue (sensitivity 80%). The addition of c-*erb*B-2 in a protocol using CEA and CA 15.3 increased the sensitivity in early diagnosis by 11.2%. This sensitivity may be obtained using c-*erb*B-2 serum levels only in patients with c-*erb*B-2 overexpression in tissue. However, the long-term benefit of early detection on therapy response and patients' survival remains to be defined.

**Acknowledgements**

This work was financed by the Ministry of Health (Fiss 96-0036). We thank Celia Aparicio and Mercedes Sasot for their excellent technical assistance.

### References

AHLEMANN LM, STAAB HJ AND ANDERER FA. (1980). Serial CEA determinations as an aid in postoperative therapy management of patients with early breast cancer. *Biomedicine*, **32,** 194–199.

ALBURQUERQUE KV, PRICE MR, BADLEY RA, JONRUP I, PEARSON D, BLAMEY RW AND ROBERTSON JFR. (1995). Pretreatment serum levels of tumour markers in metastatic breast cancer: a prospective assessment of their role in predicting response to therapy and survival. *Eur. J. Surg. Oncol.*, **21,** 504–509.

ALLRED DC, CLARK GM, MOLINA R, TANDON AK, SCHNITT SJ, GILHRIST KW, OSBORNE CK, TORMEY DC, ABELOFF MD AND MCGUIRE WL. (1992*a*). Overexpression of HER-2/neu and its relationship with other prognostic factors change during the progression of *in-situ* to invasive breast cancer. *Hum. Pathol.*, **23,** 974–979.

ALLRED DC, CLARCK GM, TANDON AK, MOLINA R, TORMEY DC, OSBORNE CK, GILHRIST KW, MANSOUR EG, ABELOFF MD AND MCGUIRE WL. (1992*b*). HER-2/neu in node-negative breast cancer: prognostic significance of overexpression influenced by the presence of *in situ* carcinoma. *J. Clin. Oncol.*, **4,** 599–605.

ALTMAN DG. (1991). *Practical Statistics for Medical Research*. Chapman-Hall: London.

CASES A, FILELLA X, MOLINA R, BALLESTA AM, LOPEZ-REVERT J AND REVERT L. (1991). Tumor markers in chronic renal failure and hemodialysis patients. *Nephron*, **57,** 183–186.

CHATAL JF, CHUPIN G, RICOLLEAU G, TELLIER JL, LE MOVEL A AND FUMOLEAU P. (1981). Use of serial carcinoembryonic antigen assays in detecting relapses in breast cancer involving high risk of metastasis. *Eur. J. Cancer*, **17,** 233–238.

COLOMER R, RUIBAL A, GENOLLA J AND SALVADOR L. (1989). Circulating CA 15.3 antigen levels in non-mammary malignancies. *Br. J. Cancer*, **59,** 283–286.

COOMBES R, POWLES T, GAZET J, FORD HT, MCKINNA A, ABBOT M, GEHRKE CN, KEYSES JW, MITCHELL PEG, PATEL S, STIMSON WH, WORWOOD M, JONES M AND NEVILLE AM. (1981). Screening for metastases in breast cancer: an assessment of biochemical and physical methods. *Cancer*, **48,** 310–315.

DAVIDSON NE AND LIPPMAN ME. (1988). Adjuvant therapy for breast cancer. In *Diagnosis and Management of Breast Cancer*, Lippman ME, Lichter AS and Danforth DN. (eds) pp. 348–387. WB Saunders: Philadelphia.

DE VITA VT Jr. (1983). The relationship between tumour mass and resistance to chemotherapy. *Cancer*, **51,** 1209–1220.

GOLDIE JH. (1989). Mathematical models of drug resistance and chemotherapy effects. In *Drug Resistance in Cancer Therapy*, Ozols RF. (ed.) pp. 13–26. Kluwer Academic: Boston.

KUFE D, INGHIRAMI G, ABE M, MAYES D, JUSTI-WHEELER H AND SCHLOM J. (1984). Differential reactivity of a novel monoclonal antibody (DF-3) with human malignant versus benign breast tumor. *Hybridoma*, **3,** 223–232.

MCKENZIE SJ, DE SOMBRE KA, BAST BS, HOLLIS DR, WHITAKER RS, BERCHUCK A, BOYER CM AND BAST RC. (1993). Serum levels of HER-2/neu (C-*erb*B-2) correlate with overexpression of p184 neu in human ovarian cancer. *Cancer*, **71,** 3942–3946.

MINTON JP, HOENH JL, GERBER DM, SHELTON J, CONNALLY DP AND SALWAN F. (1985). Results of a 400-patient carcinoembryonic antigen second-look colorectal cancer study. *Cancer*, **55,** 1284–1290.

MOLINA R AND BALLESTA AM. (1991). Evaluation of several tumor markers (MCA, CA 15.3, BCM and CA 549) in tissue and serum of patients with breast cancer. In *Breast Epithelial Antigens. Molecular Biology to Clinical Applications*, Ceriani RL. (ed.), pp. 161–168. Plenum Press: New York.

MOLINA R, FILELLA X, MENGUAL PJ, PRATS M, ZANON G, DANIELS MD AND BALLESTA AM. (1990). MCA in patients with breast cancer: correlation with CEA and CA 15.3. *Int. J. Biol. Markers*, **5,** 14–21.

MOLINA R, CIOCCA R, TANDON AK, ALLRED DC, CLARCK GC, CHAMNESS GC, GULLICK WJ AND MCGUIRE WL. (1992). Expression of HER-2/neu oncoprotein in human breast cancer. A comparison of immunohistochemical and western blot techniques. *Anticancer Res.*, **12,** 1965–1972.

MOLINA R, ZANON G, FILELLA X, MORENO F, JO J, DANIELS M, LATRE ML, GIMENEZ N, PAHISA J, VELASCO M AND BALLESTA AM. (1995). Use of serial carcinoembryonic antigen and CA 15.3 assays in detecting relapses in breast cancer patients. *Br. Cancer Res. Treat.*, **36,** 41–48.

MOLINA R, JO J, FILELLA X, BRUIX J, GIMENEZ N, CASTELLS A, HAGUE M AND BALLESTA AM. (1996). Serum levels of C-*erb*B-2 (HER-2/neu) in patients with malignant and non-malignant diseases. *Tumor Biol.*, (in press).

NARITA T, FUNAHASHI H, SATOH Y AND TAKAGI H. (1992). C-*erb*B-2 in the sera of breast cancer patients. *Br. Cancer Res. Treat.*, **24,** 97–102.

PUPA SM, MENARD S, MORELLI D, POZZI B, DE PALO G AND COLNAGHI MI. (1993). The extracellular domain of the c-*erb*B-2 oncoprotein is released from tumor cells by proteolytic cleavage. *Oncogene*, **8,** 2917–2923.

SILVESTRINI R, SANFILIPPO O AND TEDESCO G. (1974). Kinetics of human mammary carcinoma and their correlation with the cancer and the host characteristics. *Cancer*, **34,** 1252–1258.

SLAMON DJ, GODOLPHIN W, JONES LA, HOLT JA, WONG SG, KETH DE, LEVIN WJ, STUART SG, UDOBE J, ULLRICH A AND RESS MF. (1989). Studies of the HER-2 neu proto-oncogene in human breast and ovarian cancer. *Science*, **244,** 707–712.

STAAB HJ, AHLEMANN ML, KOCH HL AND ANDERER FA. (1980). Serial carcinoembryonic antigen determinations in the management of patients with breast cancer. *Oncodev. Biol. Med.*, **1,** 151–160.

VAN DALEN A. (1989). Tumor markers in breast cancer. *Ann. Ch. Gynaec.*, **78,** 54–64.

ZABRECKY JR, LAM T, MCKENZIE SJ AND CARNEY W. (1991). The extracellular domain of p185 neu is released from the surface of human breast carcinoma cell, SK-BR-3. *J. Biol. Chem.*, **266,** 1716–1720.